# Sialadenoma papilliferum-like intraductal papillary tumor with unveiling *BRAF V600E* and *PIK3CA H1047R* mutations: Case report with molecular analysis and literature review

**DOI:** 10.1016/j.ijscr.2024.109611

**Published:** 2024-04-07

**Authors:** Ziyad Alsugair, Anne Neuhart, Nazim Benzerdjeb, Anne Champagnac, Daniel Pissaloux, Aline Baltres

**Affiliations:** a Department of Pathology, Institut de Pathologie Multisite, Groupement Hospitalier Sud, Hospices Civils de Lyon, Pierre-Bénite, France; b Biopathology department, Centre Leon Berard, Lyon, France; c The Unit of Molecular Pathology, INSERM 1052, CNRS 5286 of Cancer Research Center of Lyon, Team Genetics, Epigenetics and Biology of Sarcomas, Université Claude Bernard Lyon 1, Lyon, France

**Keywords:** Sialadenoma papilliferum-like, Intraductal papillary tumor, BRAF V600E, PIK3CA, RNA sequencing

## Abstract

**Introduction:**

Sialadenoma papilliferum (SP), a rare minor salivary gland tumor, shares morphological and genetic similarities with syringocystadenoma papilliferum. Recent studies have identified BRAF V600E or HRAS mutations in SP, suggesting its neoplastic nature. Despite being uncommon, SP poses diagnostic challenges due to its resemblance to other lesions like squamous papilloma. The emergence of sialadenoma papilliferum-like intraductal papillary tumor (SP-IPT) further complicates its classification, emphasizing the need for thorough investigation.

**Case presentation:**

A 50-year-old male presented with a left palatal lesion histologically diagnosed as SP-IPT. Surgical resection revealed characteristic features, including papillary projections into cystically dilated ductal spaces. Immunohistochemistry confirmed positivity for pan-keratin AE1/AE3, cytokeratin 7, SOX10, and BRAF V600E. Whole-exome sequencing identified BRAF V600E and PIK3CA H1047R mutations. No recurrence was observed three months post-excision.

**Discussion:**

SP-IPT's diagnostic complexity stems from its resemblance to SP without an exophytic papillary component. However, shared BRAF mutations suggest a close relationship between the two entities. Similarities with skin adnexal tumors underscore the importance of molecular markers in tumor classification. The identification of PIK3CA mutation in SP-IPT adds to its molecular diversity, warranting further investigation into its clinical significance.

**Conclusion:**

This study presents a case of SP-IPT with unique histological and molecular features, highlighting its diagnostic and therapeutic challenges. The co-occurrence of BRAF V600E and PIK3CA H1047R mutations suggests a distinct molecular profile in SP-IPT, necessitating further research to elucidate its biological behavior and clinical implications.

## Highlights

• Sialadenoma papilliferum (SP) is a rare tumor accounting for only 1.1% of minor salivary gland tumors, characterized by distinct genetic alterations and a biphasic growth pattern.
• Shared mutations such as BRAF V600E or HRAS between SP and syringocystadenoma papilliferum imply a close relationship despite morphological differences.
• SP presents with both exophytic papillary and endophytic adenomatous components, often resembling other benign lesions clinically.
• A case of SP-IPT with a dual mutation of BRAF V600E and PIK3CA H1047R introducing a novel molecular insight into this rare tumor entity.

## 1 Introduction

Sialadenoma papilliferum (SP) accounts for 1.1 % of minor salivary gland tumors and for 2 % of benign tumors of these glands. Past reviews indicate that this tumor is perhaps the least common of all salivary gland tumors [1]. SP was first described by Abrams and Finck in 1969, who noted similar morphologic features with syringocystadenoma papilliferum of sweat gland origin [2]. Recent studies have also shown that these entities share similar genetic alterations (*BRAF V600E* or *HRAS* mutations) [[3], [4], [5]].

SP morphologically is characterized by a biphasic growth pattern: an exophytic papillary component and an endophytic adenomatous component [2,6,7]. There are two distinct subtypes described based on the endophytic ductal component: classic or oncocytic [3]. SP commonly occurs on the palate and clinically can be easily misdiagnosed for squamous papilloma or verrucous hyperplasia [5,8] Recently, the term “sialadenoma papilliferum-like intraductal papillary tumor (SP-IPT)” has been proposed for tumors that share morphologic characteristics with SP except for the exophytic papillary component [4]. Taking the results of molecular studies together, SP and SP-IPT share identical genetic mutations, most commonly the *B-Raf proto-oncogene, serine/threonine kinase (BRAF) V600E* mutation, which suggests their close relation. Herein we studied a case of SP-IPT with a thorough immunohistochemical and molecular investigation in order to better define the phenotype and we reviewed the literature on this subject. The case was reported in accordance with the SCARE guideline recommendation [9].

## 2 Case presentation

**Fig. 1:**
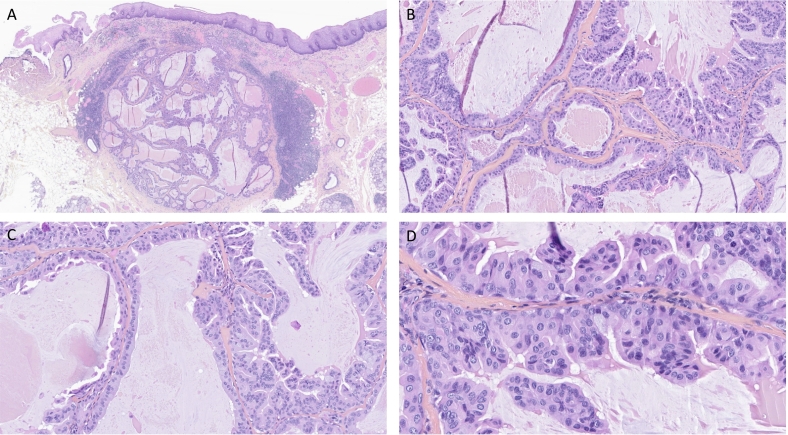
**The histological features of sialadenoma papilliferum-like intraductal papillary tumor**. (A) The lesion is well-demarcated covered by a flat surface of mucosal squamous epithelium, with a well-defined multicystic ductal proliferation observable as a submucosal nodule. (B) Papillary projections composed of cuboidal/columnar and mucous cells into the cystically dilated ductal space. (C) Branching papillary elements, consisting of two or three layers of cells, are supported by a fibrovascular connective tissue core and surfaced by columnar cells and mucous fluid. (D) The tumor cells exhibit mild to moderate nuclear atypia.

A 50-year-old male patient presented with a left palatal lesion accompanied by a painful sensation upon palpation. It was not associated with difficulty in eating nor weight loss. Radiological examination with cervico-facial and thoracic CT scans, revealed no cervical adenopathy or pulmonary lesion of secondary appearance. MRI did not find the lesion, although supra-millimetre slices were obtained. The patient did not have a prior history of trauma or surgery at this site. He underwent a successful surgical resection. A 1.5 cm resection sample with a tiny well demarcated whitish to gray lesion. The histological examination showed a well-demarcated tumor proliferation (Fig. 1A). It was formed by a cystic cavity. The tumor seems to originate from the superficial portion of salivary glands excretory ducts and characterized by papillary projections of the cuboidal/columnar and mucous cells to the cystically dilated ductal space (Fig. 1B). The cystic lumen was partially filled by many branching papillary elements, consisting of two or three layers of cells, supported by a core of fibrovascular connective tissue surfaced by columnar cells and by a mucous fluid (Fig. 1C). The lesion is surrounded by a thick and fibrous tissue wall. The tumor cells showed mild to moderate nuclear atypia (Fig. 1D), without mitoses nor necrosis. No obvious malignant transformation with invasion of surrounding tissue was detected. The lesion was covered by a flat squamous epithelium, and appeared as a sub-mucosal nodular tumor.

**Fig. 2:**
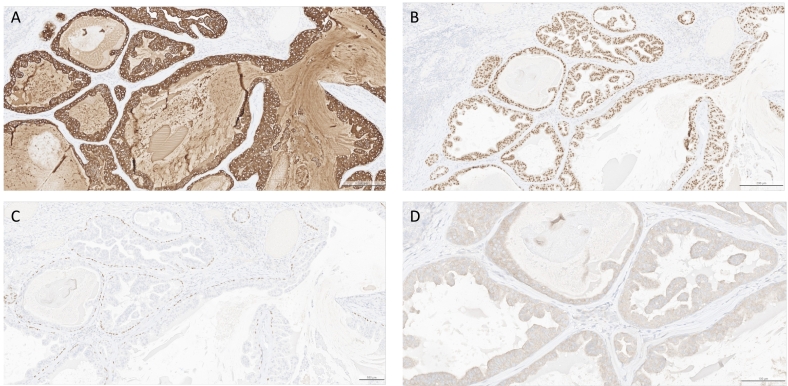
**Immunohistochemical findings**. Diffuse cellular positivity was observed for (A) cytokeratin 7 and (B) SOX10, while (C) the basal cells were marked by P63. (D) The tumor cells exhibited diffuse positivity for BRAF V600E.

On immunohistochemistry, the tumor cells demonstrated positivity for pan-keratin AE1/AE3, cytokeratin 7 (Fig. 2A), SOX10 (Fig. 2B) the basal cells were marked by P63 (Fig. 2C).

The tumor cells showed positivity of BRAF V600E (Fig. 2D) and negativity for PLAG1. The index of proliferation Ki67 was estimated at 5 %.

Whole exome sequencing identified gene mutations of *BRAF V600E* and *PIK3CA H1047R* without gene fusion including *MAML2*, *PLAG1*, *HM*GA2, *EWSR1*, *PRKD* genes.

There was no evidence of tumor recurrence eight months post-excision.

## 3 Discussion

We present a SP-IPT with its characteristic histological and molecular features in which a dual *BRAF* and *PIK3CA* mutations has been identified for the first time. These cases are usually challenging clinically and radiologcally due to their small size and anatomic localisation. These cases also are equally difficult histologically in the set of small biopsy of palatine or oral cavity lesion. The limited number of documented cases of this entity in the literature underscores the rarity of this lesion. The results of these studies are summarized in Table 1.

**Table 1:** Results from the literature of sialadenoma papilliferum-like intraductal papillary tumor.

References	Age	Sex	Site	Size (mm)	Associated mutations	Recurrence
M Nakaguro et al. 2020 [4]	80	F	Tongue	8	*BRAF V600E,* *HRAS Q61R*	NED
M Nakaguro et al. 2020 [4]	89	M	Retromolar gingiva	5	*BRAF V600E*	NED
Y Ohashi et al. 2021 [18]	52	F	Tongue	6	*BRAF V600E*	NED
S Shigemoto et al. 2022 [19]	84	F	Retromolar gingiva	15	*BRAF V600E*	NED
Current case	50	M	Soft palate	10	*BRAF V600E* *PIK3CA H1047R*	NED

F, Female; M, Male, NED: No Evidence of disease.

While these cases shared an identical morphology with SP, they lacked an exophytic papillary component. According to the WHO classification [10], these cases did not meet the histological criteria for SP. Nonetheless, the presence of *BRAF* mutations in both SP and SP-IPT suggests a potential close relationship between them. The disparity between SP and SP-IPT may be attributed to variances in their developmental stages and initial tumor proliferation site localization. A similar issue has been suggested in skin adnexal tumors such as syringocystadenoma papilliferum, tubular apocrine adenoma and papillary eccrine adenoma overlap histologically, and *BRAF* mutations are frequently observed in these three entities in 52–83 % [11,12].

Also Oh et al. [13] after a thorough review of the literature, they proposed to reconsider the nomenclature of the tubulopapillary hidradenoma-like tumor of the mandible to be classified as sialadenoma papilliferum-like intraductal papillary tumor. This suggestion based on a comprehensive comparison of its clinico-pathological characteristics, coupled with the identification of two additional cases bearing the *BRAF V600E* mutation. Table 2 summaries differential diagnosis of sialadenoma papilliferum-like intraductal papillary tumor and other analogues tumors.

**Table 2:** Common differential diagnosis of sialadenoma papilliferum-like intraductal papillary tumor and other analogues tumors.

Diagnosis	Site	Pathological findings	Common mutations
SP	Oral cavity	Exophytic squamous and inverted glandular tumor components	*BRAF V600E* *HRAS*
SP-IPT	Oral cavity	Only exophytic component	*BRAF V600E*
SCP	Skin (frequently in anogenital areas)	Exophytic and endophytic component	*BRAF V600E* *HRAS G13R*
TPH	Mandible	Glandular proliferation with apocrine and eccrine differentiation	*BRAF V600E* *KRAS*

SCP, Syringocystadenoma papilliferum; SP, Salivary sialadenoma papilliferum; SP-IPT, Salivary sialadenoma papilliferum-like intraductal papillary tumor; TPH, Tubulopapillary hidradenoma-like tumor.

The presence of *BRAF* mutations indicated the neoplastic nature of both the squamous component and the ductal component of SP. The transition of ductal epithelium to squamous epithelium seen in SP suggests that this tumor originates in the excretory ducts [4]. Furthermore, given that BRAF V600E immunoreactivity was detected in proliferative squamous and intraductal luminal cells.

Interestingly, the *PIK3CA* mutation has never been described in SP-IPT. The *PIK3CA* gene responsible for encoding the catalytic subunit of phosphoinositide 3-kinase stands out as one of the commonly mutated genes in breast carcinoma. Activating mutations in this gene have been detected in a quarter of examined cases, ranging from 8 % to 40 %. The majority of these mutations occur in specific ‘hotspot’ regions within exon 9 (helical domain) and exon 20 (kinase domain), although a smaller number of mutations have been found in other exons [[14], [15], [16], [17]].

In the present case, the PIK3CA H1047R mutation was identified in the kinase domain in exon 20, which has been frequently reported in intraductal papilloma of the breast [17]. These recent molecular insights underscore the papillomatous nature of the lesion. Despite being a benign entity, the detection of *PIK3CA* mutation in SP-IPT could potentially indicate a heightened risk of malignant transformation as was reported for breast fibroepithelial tumors. (DOI: https://doi.org/10.1038/ng.3409) This finding holds clinical relevance, suggesting the necessity of vigilant monitoring in a clinical setting.

## 4 Conclusion

This study presents a case of SP-IPT well demarcated and less aggressive with unique histological and molecular features, highlighting its diagnostic and therapeutic challenges. The co-occurrence of *BRAF V600E* and *PIK3CA H1047R* mutations suggests a distinct molecular profile in SP-IPT, necessitating further research to elucidate its biological behavior and clinical implications.

## Patient consent

Written informed consent was obtained from the patient for publication of this case report and accompanying images. A copy of the written consent is available for review by the Editor-in-Chief of this journal on request.

## Provenance and peer review

Not commissioned, externally peer-reviewed.

## Ethical approval

Ethical approval for this study was provided by the ethics committee of the medical faculty and the state medical board.

## Funding

None.

## Author contribution

ZA,AN and AB did the conception, design of the work, and the data collection; ZA, AN,NB and AB did the data analysis and interpretation; AN,NB,AC,DP did the critical revision of the article;ZA,NB and AB did the final approval of the version to be published.

## Guarantor

Ziyad Alsugair.

## Research registration number

None.

## Conflict of interest statement

The authors have declared that no competing interests exist.
